# Pancreatic atrophy caused by dietary selenium deficiency induces hypoinsulinemic hyperglycemia via global down-regulation of selenoprotein encoding genes in broilers

**DOI:** 10.1371/journal.pone.0182079

**Published:** 2017-08-01

**Authors:** Jingyang Xu, Longqiong Wang, Jiayong Tang, Gang Jia, Guangmang Liu, Xiaoling Chen, Jingyi Cai, Haiying Shang, Hua Zhao

**Affiliations:** 1 Animal Nutrition Institute, Sichuan Agricultural University, Chengdu, Sichuan, China; 2 Trace Element Research Center, Sichuan Agricultural University, Chengdu, Sichuan, China; International Nutrition Inc, UNITED STATES

## Abstract

This study was envisaged to comprehensively profile genes in selected tissues along with a few biochemical indicators and integrate resulting information with dietary selenium (Se) deficiency symptoms in broilers. A total of 120 one-day-old Cobb male broilers were equally divided into two groups and fed a Se deficient corn-soybean-based basal diet supplemented with 0.3 mg/kg sodium selenite (Control, Se adequate) or without selenite (Se deficiency) for five weeks. Effects of Se deficiency on mRNA abundance of twenty-three selenoprotein encoding genes and seventeen insulin signaling related genes were studied at day 35 in pancreas, liver and muscle along with plasma biochemical constituents and enzyme activities. Compared to healthy birds in control diet, Se deficient diet induced deficiency symptoms in 90% birds and classic nutritional pancreatic atrophy, depressed growth performance of broilers, and decreased (*P* < 0.01 to *P* < 0.05) total antioxidant capacity and activities of superoxide dismutase and glutathione peroxidase in plasma and three other tissues. Se deficiency resulted in 58% higher mortality than control birds. Dietary Se deficiency down-regulated (*P* < 0.01–0.05) eighteen selenoprotein encoding genes in pancreas, fourteen genes in muscle and nine genes in liver, and up-regulated (*P* < 0.05) *Txnrd1* and *Selx* in liver. Meanwhile, six, thirteen and five insulin signaling related genes were down-regulated (*P* < 0.01–0.05) in pancreas, muscle and liver, respectively, and three genes were up-regulated (*P* < 0.01) in liver. The decrease (*P* < 0.05) in levels of plasma insulin, total triglyceride and total cholesterol, and concurrent elevated (*P* < 0.05) levels of plasma glucose and inflammatory cytokines accompanied the global down-regulation of selenoprotein encoding- and insulin signaling related- genes in Se deficient birds. It was concluded that dietary Se deficiency induces nutritional pancreatic atrophy and metabolic disorder of glucose and lipid in broilers via down-regulation of selenoprotein encoding- and insulin signaling related- genes, indicating potential roles of these genes in metabolic regulation.

## Introduction

Selenium (Se) is an essential micronutrient in animals and human that has been shown to exert its important biological functions through selenoproteins [1, 2]. Dietary Se supplementation invokes the prevention of certain forms of oxidative stress-related chronic diseases, cardiovascular diseases and diabetes [3]. Although its importance for the prevention of certain forms of diseases such as exudative diathesis and nutritional pancreatic atrophy in chicken [4], Keshan disease and muscular dystrophy in human [5], and muscle malnutrition in swine [6] has been well established, the association between Se and glucose metabolism remains unclear. Recent studies report that supranutritional Se intake is associated with development of insulin resistance and hyperinsulinemia, and the alteration in blood lipid profiles in human [7, 8], rodent [9], pig [10] and chicken [11] models. However, a number of animal experiments and epidemiologic investigations have shown the correlation between Se deficiency and glucose or lipid metabolic impairment [4, 12] and as insulin mimetic, Se can activate key proteins involved in the insulin signaling pathway and has an anti-diabetic effect [13]. Studies in mice show that selenoprotein deficiency leads to elevated fasting plasma glucose levels in mutant selenocysteine tRNA transgenic mice [14]. Selenate shows regulatory effects on glycolytic, gluconeogenic and fatty acid metabolism pathways which are disturbed in diabetic disorders [15] and sodium selenite stimulates glucose uptake in cultivated adipocytes and dissected skeletal muscle of rats [16, 17]. Both insulin signaling and insulin secretion are linked to the cellular redox state [18–20], providing a rationale for an interference of Se and antioxidant selenoproteins with insulin regulated metabolic pathways. Se deficiency results in exudative diathesis, pancreatic atrophy [4] and changes of histomorphology and cell structure of pancreas [21] in chicken, which indicates that Se is a key factor to ensure normal functioning of the pancreas, as well the synthesis and function of insulin.

Moreover, pancreas, liver and muscle are the main organs related to carbohydrate and lipid metabolism. Pancreas is responsible for the secretion of insulin synthesis and secretion which are regulated by transcriptional factors, signaling molecules, and functional proteins encoded by genes such as pancreatic and duodenal homeobox 1 (Pdx1) [22], neurogenic differentiation 1 (Neurod1) [23], hepatocyte nuclear factor 1 homeobox A (Hnf1a) [24], forkhead box A2 (Foxa2) [25] and glucose transporter 2 (Glut2). Insulin secreted by pancreatic β-cells stimulates glycogen synthesis and decreases glucose production in the liver [26], and increases glucose uptake, utilization and storage in fat and muscle [27]. Key proteins such as insulin receptor (Ir), insulin substrate 1 (Irs1), insulin substrate 2 (Irs2), serine/threonine protein kinase 1 (Akt1), forkhead box O 1 (Foxo1), phosphoinositide 3-kinase (Pi3k) are relating to insulin signaling cascade in insulin target tissues. The deviant expression of these proteins may lead to disruption on carbohydrate and lipid metabolism in animal [28]. Our previous study has indicated the presence of correlations between the expression of insulin signaling related genes and Se through redox system or other ways [11].

It is well established that Se plays important biological roles in living organisms, mostly through its incorporation into a family of proteins called selenoproteins, and the incorporated amino acid selenocysteine is considered the 21^st^ amino acid [29]. The importance of Se to protect metabolic processes from reactive oxygen species accumulation *in vivo* is characterized by its role as a constituent of antioxidant defense systems [30]. It is likely that the insulin regulated glucose and lipid metabolism by Se is partly rooted in selenoproteins [31]. Selenoproteins play important physiological roles in animal metabolism and is involved in the distal signaling of the insulin signaling cascade, by regulating glycolysis, gluconeogenesis, fatty acid synthesis and the pentose phosphate pathway [32]. Dietary Se deficiency results in pancreas lesions in chicken, which can be ameliorated or cured by Se supplementation [4], indicating an essential role of Se in pancreas. However, the effects of dietary Se deficiency on expression of selenoprotein encoding- and insulin signaling related- genes in chicken remain unclear, and the linkage between expression profiles of selenoprotein gene and metabolic impact of dietary Se deficiency needs to be established. Therefore, we determined the impact of dietary deficiency of Se (induced by feeding Se-deficient diets, see materials & methods) on plasma biochemical, antioxidant antributes and inflammatory cytokines measurements along with expression of selenoprotein encoding- and insulin signaling related- genes in liver, muscle, and pancreas of chicken.

## Materials and methods

### Broilers and diets

The animal protocol was approved by Animal Care Office of Sichuan Agricultural University, Chengdu, China and the animal experimental procedure was performed according to the guidelines for the care and use of experimental animals established by the Ministry of Agriculture of People’s Republic of China. A total of 120 one-day-old male Cobb broilers (Wenjiang Zhengda Poultry, Sichuan province) were allotted into two dietary treatment groups (*n* = 60/group). Selenium deficiency was induced in broilers (Se deficiency group) by feeding a low-Se corn-soybean-based practical diet (basal diet) (S1 Table) formulated from the low Se containing feed ingredients that were grown on Se deficient areas in southwest of China. The control (Se-adequate group) birds were fed basal diet supplemented with 0.3 mg/kg sodium selenite (provided by Chelota group). The analyzed Se concentration in basal diet was below 0.02 mg Se/kg of feed (as fed basis). Chicks were housed in cages with electrically heated units and the temperature was maintained at 32, 28, and 25 ^o^C for the first, second, and subsequent weeks, respectively. The birds were free access to feed and distilled water. The experiment lasted for five weeks. Individual body weights of birds were measured weekly. General health, clinical symptoms of Se deficiency diseases, and mortality were recorded daily.

### Sample collection and preparation

At the end of the experiment, birds were fasted for eight hours, and then six birds per group with average body weight (1.19 ± 0.09 kg in Se deficiency group vs 1.38 ± 0.08 kg in control group) were selected and sacrificed to collect blood, liver, pectoral muscle and pancreas samples. After immediate dissection on an ice-cold surface, the organs were rinsed with ice-cold sterile deionized water, and divided into aliquots using the surgical scissors. The tissue samples were snap-frozen in liquid nitrogen, and stored at -80 ^o^C until use. Part of pancreas samples were fixed in 10% buffered formaldehyde for pathological observation at the same time. Plasma samples were prepared by centrifugation of the whole blood (sodium ethylenediaminetetraacetic acid (EDTA) as anticoagulant, 2000×g for 15 min, 5804R Centrifuge, F45-30-11 rotor, Eppendorf) and stored at -80 ^o^C.

### Pathological observations

Exudative diathesis was identified based on the gross appearance and confirmed by autopsy [33, 34]. For histopathology, pancreas samples were embedded in paraffin wax, cut into 5 μm thin slices, mounted onto slides, and stained with hematoxylin and eosin (H&E) according to the conventional histology methods. Histological sections were examined and photographed using a Nikon (TS100) light microscope and pathological changes in pancreas were recorded.

### Biochemical assays

Selenium concentration in feed and tissues were measured using the hybrid generation-atomic fluorescence spectrometer (AFS-3200, Titan instrument) against the standard Se reference [GBW (E) 08044, National Research Centre for Certified Reference Materials, Beijing, China]. Plasma glucose, insulin, total triglyceride (TG), total cholesterol (TC), interleukin 6 (IL-6) and tumor necrosis factor-α (TNF-α) concentrations were measured using corresponding assay kit (Jiancheng Bioengineering, Nanjing, China) according to the manufacturer’s instructions. Tissues samples (around 0.5 g) were thawed on ice, homogenized in nine folds ice-cold isotonic saline (0.9% NaCl) using an Ultra-Turrax homogenizer at 7,000×g for 15 s, centrifuged at 1,000×g for 10 min at 4°C. The supernatant was then collected for subsequent biochemical analysis.

Total antioxidant capability (T-AOC), malondialdehyde (MDA), activity of superoxide dismutase (SOD) and glutathione peroxidase (GSH-Px) were measured using corresponding assay kits (Jiancheng Bioengineering, Nanjing, China). Concentration of protein was determined with bicinchoninic acid (BCA) method using a commercial BCA protein assay kit (Jiancheng Bioengineering, Nanjing, China). The optical density (OD) values were measured with an ultraviolet-visible spectrophotometer (Model 680, Bio-rad, Hercules, CA, USA). For each measurement, the compared samples were run on the same plate to eliminate potential errors origin from different plates.

### Q-PCR analyses of mRNA abundance

The primers (S2 Table) for the twenty-three selenoprotein encoding genes, seventeen insulin signaling related genes, and two reference genes: β-actin (*Actb*) and glyceraldehyde 3-phosphate dehydrogenase (*Gapdh*), were designed using Primer Express 3.0 (Applied Biosystems, Foster City, CA). RNA extraction, quality control, real-time quantitative PCR procedure, and relative mRNA abundance quantification were performed as described in previous studies [10, 35, 36] with the exception that the total RNA of pancreas was extracted using RNeasy Tissue Mini Kit (Takara, Dalian, China). The qPCRs were performed on the ABI 7900HT system (Applied Biosystems, Foster City, CA) in a final volume of 10 μL using Qiagen one-step RT-PCR kit (Qiagen, Duesseldorf, Germany), 100 ng total RNA in each sample was applied. Melting curve and cycle threshold analyses were performed to confirm the specificity of amplification. The relative mRNA abundance quantification was the same as previously described by our group using the 2^-ΔΔCt^ method [35, 36]. For each of the target gene in a given tissue, all samples were run on the same 384-well plate (Applied Biosystems, Foster City, CA).

### Statistical analysis

Independent *t*-test (SPSS for Windows 13.0; Chicago, IL, USA) was used to examine effects of the dietary Se deficiency on biochemical constituents, enzyme activities and mRNA levels of each gene within a given tissue. Data are presented as means ± SE and significance level was set at *P* ≤ 0.05 unless indicated otherwise.

## Results

### Growth performance and Se deficiency symptoms

**Fig 1:**
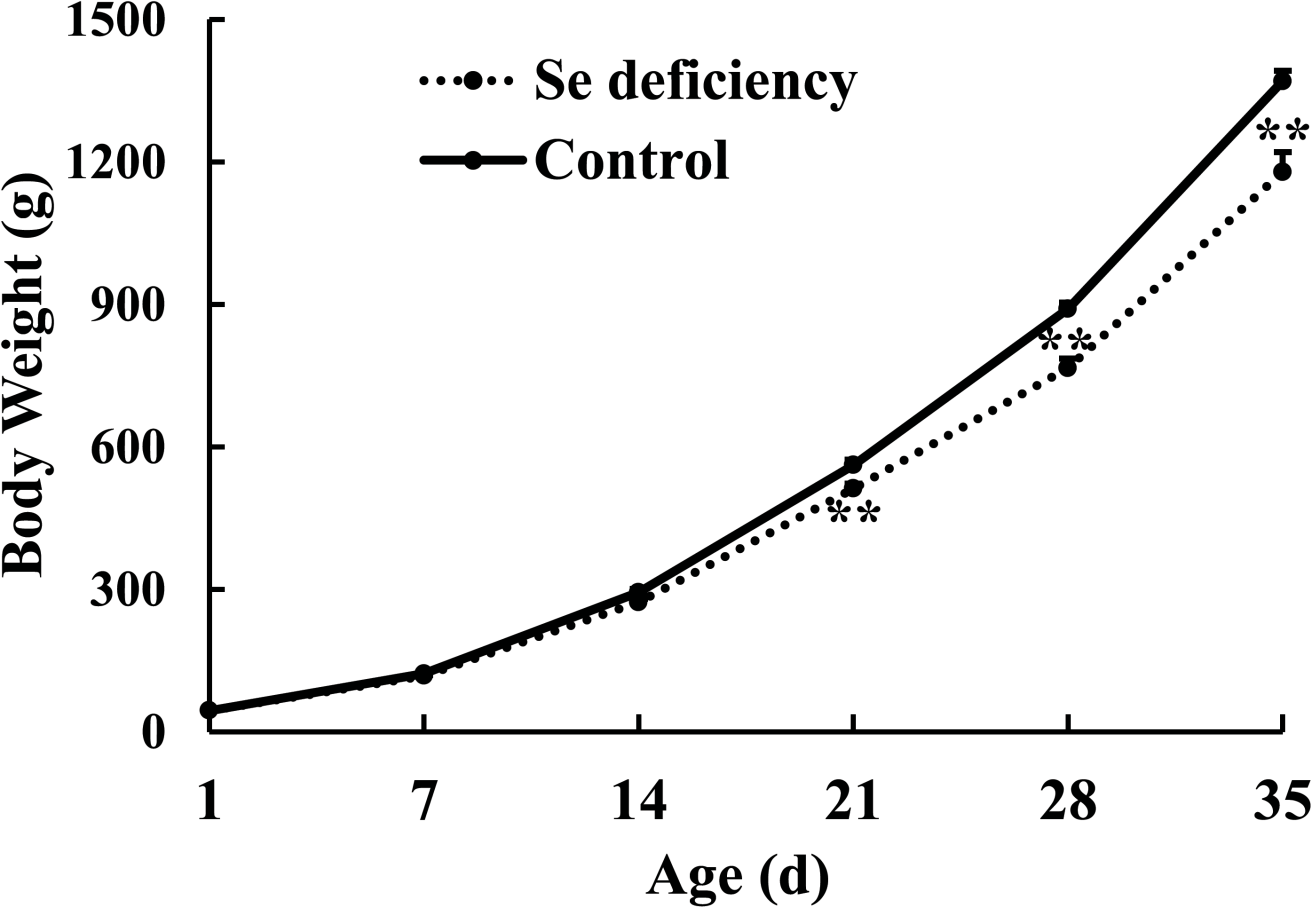
Effects of dietary Se deficiency on body weight of broiler for 5 week. Data are presented as means ± SE (*n* = 11–60). **P < 0.01.

Dietary Se deficiency suppressed growth performance of broilers (Fig 1). Compared to the Se-adequate control group (0.3 mg Se/kg), dietary Se deficiency decreased (*P* < 0.01) body weight of broiler at the third, fourth and fifth weeks.

Birds of Se deficient group displayed classic Se deficiency clinical symptoms characterized by edema or cyanotic patches in ventral parts of body such as breast, under the wings, ventral surface of the face, down the legs, abdomen and mandibular space. The neck appeared pendulous giving soft feel on palpation. Other symptoms included depression, poor feather condition, swollen legs, difficulty in standing and walking, uncoordinated movement and loss of appetite (S1 Fig). The birds with exudative diathesis died within 24–72 hours, mostly in night hours, but noticed next day morning.

**Fig 2:**
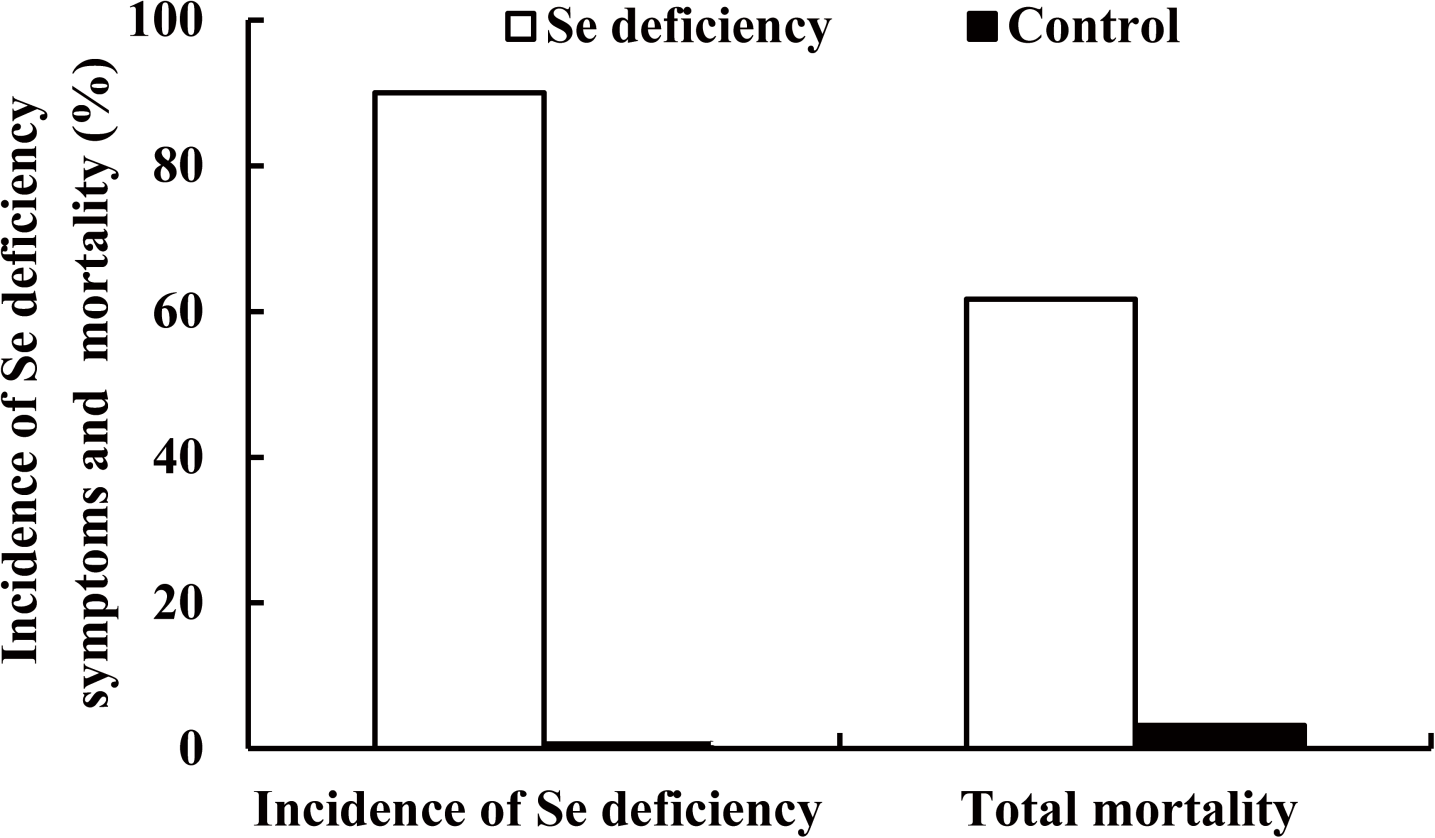
Incidence of Se deficiency symptoms and cumulative mortality in broilers fed Se deficient diets over five week period. Figures on incidence of Se deficiency symptoms and cumulative/total mortality in birds are expressed in percentage.

Week-wise data on Se deficiency symptoms and mortality in broilers fed Se deficient diet over five weeks is given in Table 1, while cumulative figures (%) on deficiency and mortality is given in Fig 2. Se deficiency symptoms first appeared in the fourth week, and of the total 90% birds that showed deficiency symptoms (Fig 2), 40% i.e. twenty-four birds showed symptoms in fourth week, while rest 50% showed in fifth week (Table 1). Similarly, death in birds due to Se deficiency also first appeared in the fourth week, and of the total 61.7% mortality (Fig 2), 23.3% i.e. fourteen birds died in fourth week, while rest 38.3% died in the fifth week (Table 1).

**Table 1:** Week-wise incidence of Se deficient symptoms and mortality in broilers fed on Se deficient diet over five weeks.

Item	Se deficiency	Control
Week	Number of birds with Se deficiency symptoms	
1^st^, 2^nd^ and 3^rd^	0	0
4^th^	24	0
5^th^	30	0
	Number of birds died	
1^st^ and 2^nd^	0	0
3^rd^	0	1
4^th^	14	0
5^th^	23	1

### Pathological changes

**Fig 3:**
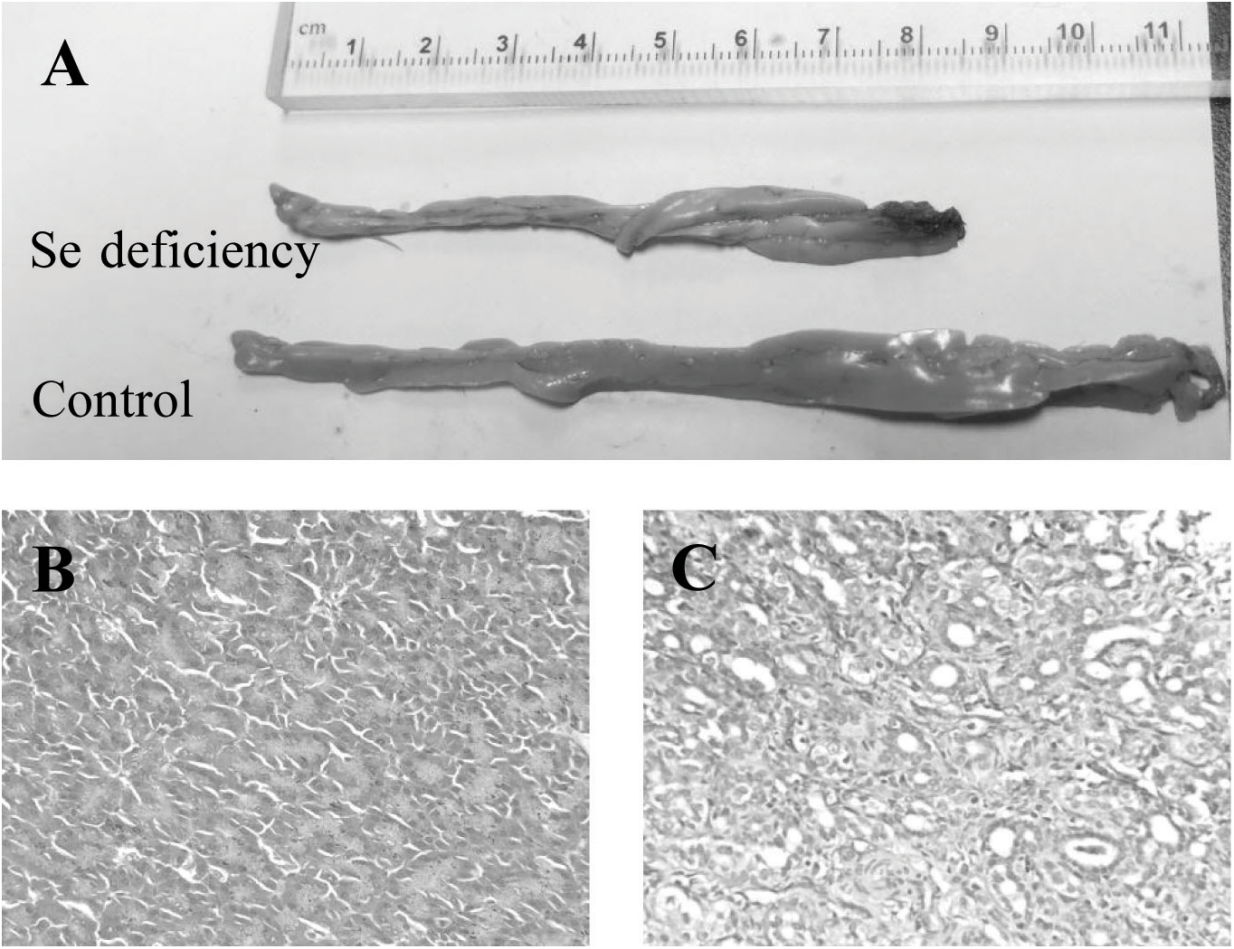
Comparison of the pancreas of broiler fed on Se deficient and control diet at fifth week. Gross morphometry (A), and histopathological view: control group (B) and Se-deficient group (C).

At the end of experiment in fifth week, autopsy of chicken with exudative diathesis (S2 Fig) showed the typical greenish, gelatinous edema, with subcutaneous (under the skin) hemorrhage in the ventral parts of body invariably the breast muscle, besides other areas such as underneath the wings, the ventral side of the face, and down the legs. Gross pathological changes in the target organ, pancreas, revealed that dietary Se deficiency exhibited negative impact on development of pancreas in chicken and resulted in pancreatic atrophy at the end of experiment (Fig 3). Gross morphometric measurements showed that Se deficiency resulted in a shorter (*P* < 0.01) length of pancreas (8.46 ± 0.47 cm) compared to those fed the control diet (11.40 ± 0.22 cm) (Fig 3A). Five weeks of Se deficiency induced pathological lesions in pancreas. Compared to the pancreas from chicken fed the control diets (Fig 3B), pancreas of birds fed the Se deficient diet exhibited appearance of large amounts of marked fibrous tissues, intracellular vacuolation and hyaline body formation (Fig 3C), the loss of zoning in the cells and considerable shrinkage of the acini in size, fibrous tissue replacement, disappeared pancreatic acinar structure, and hyperplasia of the connective tissue that formed the grid (Fig 3C).

### Tissue Se concentration, plasma biochemical attributes and inflammatory cytokine measurements

Compared with control group, dietary Se deficiency significantly decreased (*P* < 0.01) concentration of Se in plasma and liver of broilers by 79.5% and 73.1%, respectively (Table 2). Dietary Se deficiency elevated (*P* < 0.05) the fasting plasma glucose concentration and decreased (*P* < 0.05) plasma insulin in birds (Table 2). Se deficiency resulted in decreases (*P* < 0.01) in concentrations of TG and TC by 61.2% and 45.1%, respectively (Table 2). Likewise, dietary Se deficiency also exhibited negative impact on plasma inflammatory cytokines. Birds fed the Se deficient diet had plasma concentrations of IL-6 and TNF-α increased by 40.4% and 23.4% (*P* < 0.05) compared to the control group, respectively (Table 2).

**Table 2:** Effects of dietary Se deficiency on liver Se deposition, plasma biochemical attributes and inflammatory cytokine at fifth week.

Item	Se deficiency	Control	*P* value
Plasma Se (μmol/L)	0.63±0.09	3.08±0.15	<0.01
Liver Se (μmol/kg)	2.40±0.25	8.91±0.75	<0.01
IL-6 (pg/mL)	254.04±21.53	180.92±14.67	<0.05
TNF-α (pg/mL)	17.94±0.45	14.54±1.14	<0.05
TG (mmol/L)	1.12±0.30	2.89±0.44	<0.01
TC (mmol/L)	2.54±0.27	4.63±0.15	<0.01
Insulin (IU/L)	6.27±0.92	9.14±0.43	<0.05
Glucose (mmol/L)	19.43±3.80	6.01±0.40	<0.05

Values are expressed as means ± SE, *n* = 6.

Abbreviations: IL-6- Interleukin6; TNF-α- Tumor necrosis factor- α; TG- Total triglycerides; TC- Total cholesterol

### Antioxidant attributes in plasma and tissues

The results on effects of dietary Se deficiency on antioxidant attributes are shown in Table 3. Compared to the control group, dietary Se deficiency decreased (*P* < 0.01) activity of GSH-Px and T-AOC by 76.5% and 56.6% in plasma, respectively, and decreased (*P* < 0.01–0.05) activities of GSH-Px by 58.0%, 73.6% and 69.0% in liver, muscle and pancreas, respectively. Dietary Se deficiency exhibited negative impact on activity of SOD, which were decreased (*P* < 0.01) by 24.2% and 31.9% in liver and pancreas, respectively. In liver, dietary Se deficiency also resulted in decreased (*P* < 0.05) T-AOC by 18.9% and increased (*P* < 0.05) concentration of MDA by 82.3%.

**Table 3:** Effects of dietary Se deficiency on antioxidant attributes measurements in plasma, liver, muscle and pancreas at fifth week.

Item	Se deficiency	Control	*P* value
Plasma (U/ml)			
GSH-Px	166.43 ± 5.63	706.36 ± 64.62	<0.01
SOD	14.34±1.70	16.53±0.90	0.28
MDA	0.72±0.08	0.63±0.05	0.38
T-AOC	2.29±0.19	5.28±1.11	<0.05
Liver (U/mg prot)			
GSH-Px	4.24±1.01	10.10±1.09	<0.01
SOD	120.92±10.0	159.52±6.52	<0.01
MDA	0.62±0.09	0.34±0.03	<0.05
T-AOC	0.73±0.03	0.90±0.06	<0.05
Muscle (U/mg prot)			
GSH-Px	1.99±0.48	7.53±1.69	<0.05
SOD	139.84±10.60	160.24±16.16	0.32
MDA	0.41±0.04	0.34±0.02	0.16
T-AOC	0.36±0.05	0.34±0.03	0.75
Pancreas (U/mg prot)			
GSH-Px	8.17±1.82	26.37±3.22	<0.01
SOD	12.23±1.54	17.96±1.08	<0.01
MDA	0.26±0.07	0.17±0.05	0.36
T-AOC	0.35±0.14	0.48±0.11	0.46

Values are expressed as means ± SE, *n* = 6.

Abbreviations: T-AOC: Total antioxidant capability; MDA- Malondialdehyde; SOD-Superoxide dismutase; GSH-Px- Gutathione peroxidase.

### mRNA abundances of selenoprotein encoding genes

**Fig 4:**
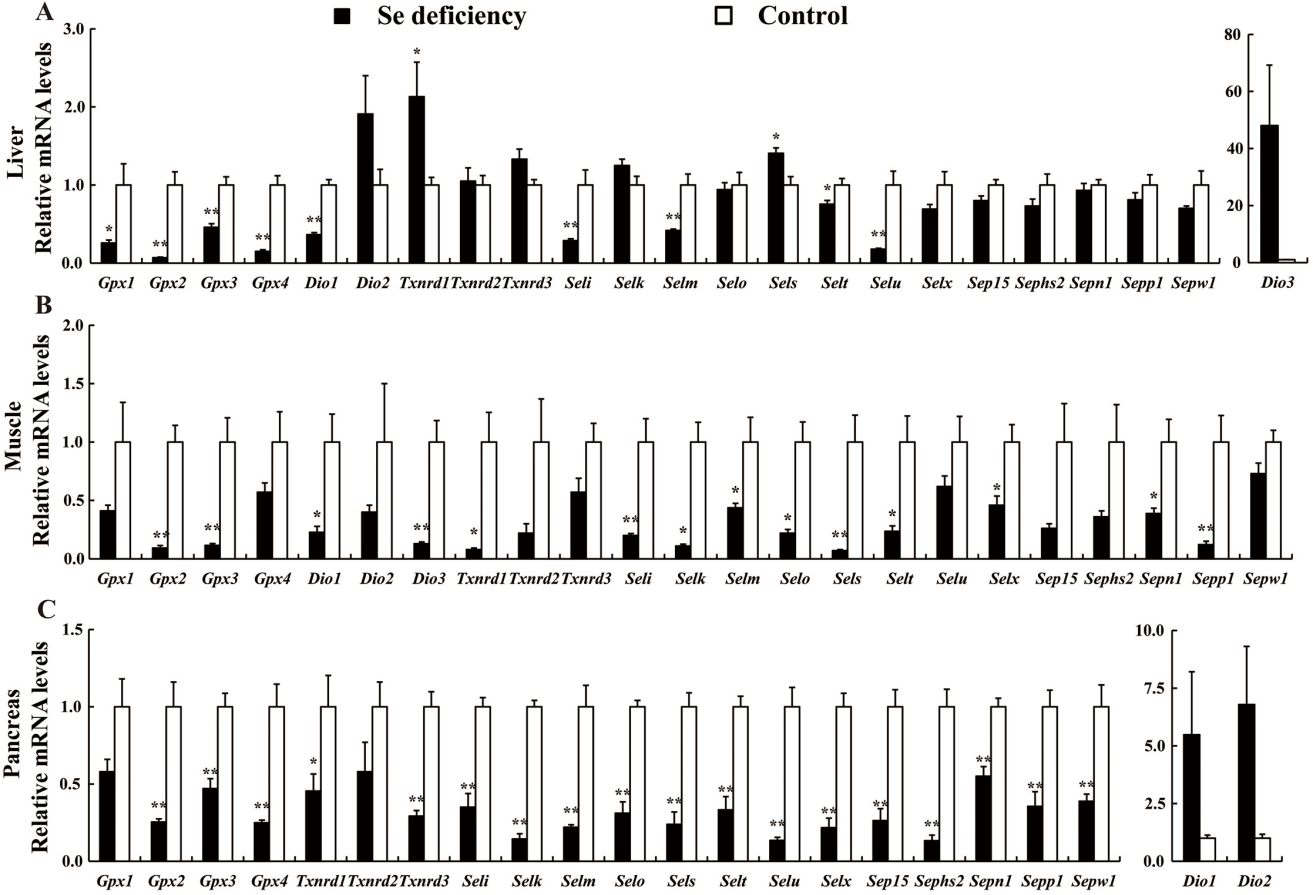
**Effects of dietary Se deficiency on relative mRNA levels of the selenoprotein encoding genes in liver (A), muscle (B) and pancreas (C) of chickens compared with those fed the control diet at fifth week**. Data are presented as means ± SE (*n* = 6). Asterisks indicate different from control: *P < 0.05, **P < 0.01.

Expression of twenty-three selenoprotein encoding genes in liver, muscle and pancreas at the fifth week showed three patterns in response to dietary Se deficiency (Fig 4). The first pattern was that dietary Se deficiency decreased (*P* < 0.01–0.05) mRNA levels in the three tissues compared with the control group fed on 0.3 mg/kg Se. This down-regulation included nine genes (*Gpx1*, *Gpx2*, *Gpx3*, *Gpx4*, *Dio1*, *Seli*, *Selm*, *Selt* and *Selu*) in liver (Fig 4A), fourteen genes (*Gpx2*, *Gpx3*, *Dio1*, *Dio3*, *Txnrd1*, *Selk*, *Seli*, *Selm*, *Selo*, *Sels*, *Selt*, *Sepn1*, *Sepp1* and *Selx*) in muscle (Fig 4B), and eighteen genes (*Gpx2*, *Gpx3*, *Gpx4*, *Txnrd1*, *Txnrd3*, *Seli*, *Selk*, *Selm*, *Selo*, *Sels*, *Selu*, *Selt*, *Sep15*, *Sepn1*, *Sepp1*, *Sepw1*, *Selx* and *Sephs2*) in pancreas (Fig 4C). The second pattern was manifested as higher (*P* < 0.05) mRNA levels in tissues of birds fed the Se deficient diet than those in birds fed the control diet. Among twenty-three selenoprotein encoding genes, only two selenoprotein encoding genes (*Txnrd1* and *Sels*) were up-regulated in liver (Fig 4A) by Se deficient diet. Thirdly, dietary Se deficiency did not influence expression of selenoprotein encoding genes in the three tissues. Individual gene specific expression level in a particular tissue and its significance (*P* < 0.05 or *P* < 0.01) for all twenty-three genes is indicated in accompanying Fig 4.

### mRNA abundances of insulin signaling related genes

**Fig 5:**
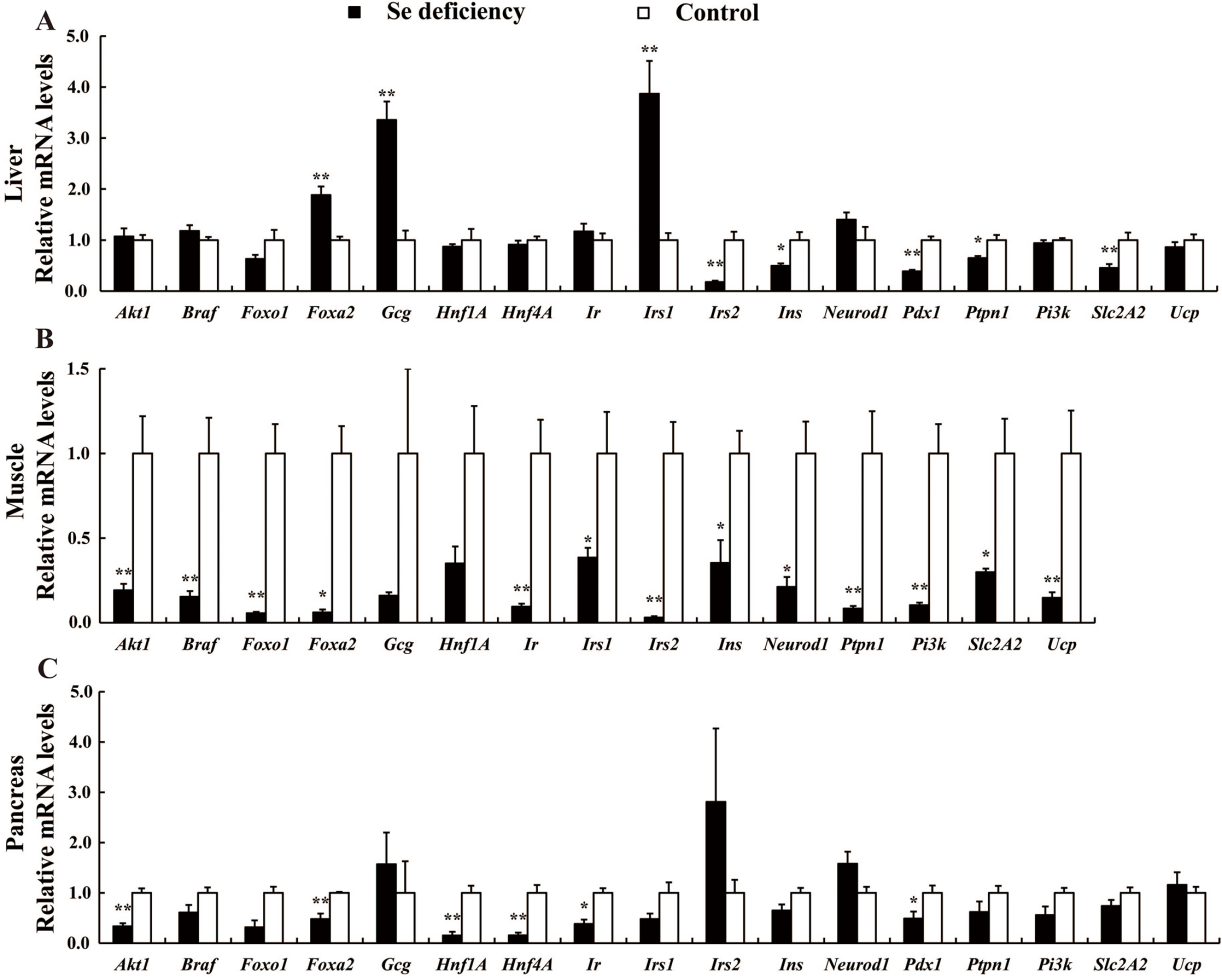
**Effects of dietary Se deficiency on relative mRNA levels of the insulin signaling related genes in liver (A), muscle (B) and pancreas (C) of chickens compared with those fed the control diet at fifth week**. Data are presented as means ± SE (*n* = 6). Asterisks indicate different from control: *P < 0.05, **P < 0.01.

Among the seventeen insulin signaling related genes, five genes (*Irs2*, *Ins*, *Pdx1*, *Ptpn1* and *Slc2A2*) in liver (Fig 5A), thirteen genes (*Akt1*, *Braf*, *Foxo1*, *Foxa2*, *Ir*, *Irs1*, *Irs2*, *Ins*, *Neurod1*, *Ptpn1*, *Pi3k*, *Slc2A2* and *Ucp*) in muscle (Fig 5B), and six genes (*Akt1*, *Foxa2*, *Hnf1A*, *Hnf4A*, *Ir* and *Pdx1*) in pancreas (Fig 5C), were down-regulated (*P* < 0.01–0.05) subjected to the Se deficient diet. In contrast, only three genes (*Foxa2*, *Gcg* and *Irs1*) (Fig 5A) were up-regulated (*P* < 0.01) in liver by Se deficiency. Of all the genes assayed, some genes provided results that were too close to background to be interpreted or reported, these including *Dio3* in pancreas, *Hnf4A* and *Pdx1* in muscle. Expression of each gene in a particular tissue and its significance (*P* < 0.01–0.05) for all seventeen insulin signaling related genes is indicated above bar in Fig 5.

## Discussion

This study comprehensively profiled Se deficiency in chickens. We convincingly demonstrated replication of classical Se deficiency disease, nutritional pancreatic atrophy, in broilers fed on a practical corn-soy diet formulated from ingredients obtained from the Se deficient areas of China. Dietary Se deficiency elevated fasting plasma glucose and reduced plasma insulin of chicken, which was associated with the alteration of mRNA expression of seventeen insulin signaling related genes in three tissues. In present study, dietary Se deficiency suppressed growth performance **(**Fig 1**)** and induced Se deficient symptoms (S1 Fig) including exudative diathesis (S2 Fig) and pancreatic lesions in chicken **(**Fig 3**)**. Such classical Se deficiency symptoms were consistent with previous studies in chicken fed Se deficient amino acid diet [21, 33, 34,37]. Unlike present study, in a recent report, chicken fed Se deficient diet (< 0.02 mg/kg) exhibited no mortality and no gross pancreatic abnormalities [38], however the diets employed in this study contained 15 times higher vitamin E than normal requirement. There are some well-established facts on interrelationships between vitamin E and Se. First, diets supplied with five times of vitamin E than normal requirement alleviate, but not prevent, the Se deficient symptoms [39]. Secondly, pancreatic atrophy in the chicken can be prevented by Se, while vitamin E supplementation reduces the biomarker of Se deficiency [37]. Earlier reports have decoded some nutritional effects of Se deficiency on pathogenesis of exudative diathesis and pancreatic atrophy in chicken using synthetic amino acid diets [40, 41]. Replication of the classical Se deficiency symptoms by practical diets provided us chance to unravel potential molecular mechanisms related to the development of this pathogenesis and the role of Se. In this study, dietary Se deficiency decreased Se concentrations in plasma and liver **(**Table 2**)** and lowered plasma GSH-Px activity **(**Table 3**)** in broilers, which was similar to those in rodents [14], pigs [10, 35] and chicken [38, 39, 42]. Dietary Se deficiency (< 0.02 mg Se/kg) elevated fasting plasma glucose and decreased plasma insulin in birds (Table 2) while it exhibited no effects on fasting plasma glucose and insulin concentration in pigs [10, 35] and rat dams [9] models. Selenium is involved in carbohydrate and lipid metabolism in animal [11, 14] and high Se exposure is associated with increased plasma lipid levels [11]. In present study, dietary Se deficiency induced pancreatic atrophy was associated with hypoinsulinemic hyperglycemia, and chicken also exhibited lower plasma TG and TC concentrations **(**Table 2**)**. Therefore, Se deficiency and excess had different impact on carbohydrate and lipid metabolism.

Selenium is an important integral component of the antioxidant enzyme GSH-Px and it is actively involved in the antioxidant defense systems [43] along with SOD. Together with Vitamin E, it helps maintain membrane stability via maintining the redox state of a cell and preventing oxidative damage to membrane. We compared the effects of dietary Se deficiency on antioxidant attributes and the results showed that the Se deficient diet decreased T-AOC in plasma and liver, GSH-PX in plasma, liver, muscle and pancreas, and SOD in liver and pancreas of broilers **(**Table 3**)**, indicating eminent membrane lipid peroxidation as revealed by increased MDA value in the liver. The membrane damage causes beginning of the extravasation and finally edema. This is consistent with previous studies [37, 41]. GSH-Px and SOD are important intracellular antioxidant enzymes. Among different forms of Se-dependent GPXs, GPX1 is the first identified and the most abundant selenoprotein, which accounts for over 90% of the total GPX activity in the most of tissues [44]. The SOD catalyzes the dismutation of superoxide into oxygen and hydrogen peroxide. Previous studies indicate the correlation between plasma glucose metabolism and intracellular antioxidant enzymes, and overexpression of antioxidant selenoproteins results in hyperinsulinemia with decreased insulin sensitivity [7, 45]. Under Se deficient condition, the lower expression of antioxidant enzymes (Table 3) was associated with elevated plasma glucose and decreased plasma insulin (Table 2). Selenuim exerts most of its known biological functions through selenocysteine-containing selenoproteins, many of which are antioxidant enzymes [3, 46]. Therefore, the observed changes in plasma insulin, lipid, tissue antioxidant enzyme activities and expression of insulin signaling related genes in present study indicated that dietary Se deficiency may affect insulin metabolism via changes in expression of selenoprotein encoding genes, which may contribute to the development of Se deficient symptoms such as pancreatic atrophy in broilers.

To unravel the interference of selenoproteins with the insulin regulated carbohydrate and lipid metabolism, we investigated the effects of dietary Se deficiency on mRNA expression of selenoprotein encoding genes in three organs of broiler. The results showed that dietary Se deficiency globally down-regulated selenoprotein encoding genes in these tissues, including eighteen genes in pancreas, fourteen genes in muscle and nine genes in liver **(**Fig 4). This is consistent with previous studies [38, 39, 42, 47, 48], which reported gene expression in birds fed low-Se diets (0.014–0.03 mg Se/kg diet) versus birds supplemented with 0.15–0.3 mg Se/kg. Selenoprotein transcripts were found to be globally down-regulated in liver [39], muscle [39, 47] and pancreas [48] due to Se deficiency. Pancreas is the organ with high redox level and low amounts of the major antioxidant enzymes in maintaining its insulin secretion function [49], and muscle and liver are the important insulin target organs. Among the down-regulated selenoprotein encoding genes, *Gpx1* plays a key role in glucose homoeostasis in mice [14] and myocytes [50]. The down-regulation of *Gpx* family genes in pancreas, liver and muscle was consistent with decreased activity of GSH-Px in plasma and three organs. Research from 1990s show that loss of pancreatic GPX activity is often associated with islet dysfunction [51]. Intriguingly, pancreatic islet cells produce a relatively low amount of antioxidant enzymes including GPx1 and SOD [52] and β cells are susceptible to oxidative stress that can be induced by hyperglycemia. *Dio1* and *Seli* may be involved in lipid metabolism [36, 53], thus, the decreased expression of these genes in liver and muscle might partially contribute to the lower plasma TG and TC. Se deficiency down-regulated *Txnrd1* in muscle, *Txnrd1* and *Txnrd3* in pancreas **(**Fig 4**)**, which potentially mediates thioredoxin activity and control the redox state [54]. Moreover, *Selk*, *Selo*, *Slem*, *Sepn1* and *Sepw1* are all participated in redox in cells [55–58]. These global down-regulations of selenoprotein encoding genes might reflect a general adverse oxidative effect of the Se deficiency on tissue redox control. Proper regulation of the cellular redox balance is of prime importance to maintain and adapt pancreatic insulin secretion and to promote the insulin sensitivity of target tissues [45]. The decreased expression of antioxidative selenoprotein encoding genes might disturb the balance between oxidant and antioxidant agent and induce oxidative stress and even damage, thus impaired the pancreatic insulin secretion and function, induced hypoinsulinemia and hyperglycemia **(**Table 2**)**. Se deficiency led to the down-regulation of *Sepp1* in pancreas and muscle **(**Fig 4**)**, which is associated with the regulation of glucose metabolism in human or mammal model [59]. Among the twenty-three selenoprotein encoding genes, only *Txnrd1* and *Sels* were up-regulated in liver **(**Fig 4A**)**. Up-regulation of SelS, a component of endoplasmic reticulum protein degradation, enhances pro-inflammatory cytokine expression [60], which may partly explain that dietary Se deficiency elevated IL-6 and TNF-α in plasma **(**Table 2**)**. The up-regulation of *Txnrd1* in liver was consistent with previous study in chicken [39], however, its significance is still unclear. As mentioned above, dietary Se deficiency globally down-regulated selenoprotein encoding genes in three tissues and pancreas had the most numbers of affected genes **(**Fig 4**)**. In contrast, less selenoprotein encoding genes were affected by dietary Se in liver or muscle in previous studies in the pig [10, 35] or rodent [61] models, indicating broilers are more susceptible to dietary Se deficiency [39]. Therefore, the unique response of selenoprotein encoding genes to dietary Se deficiency in these insulin sensitive tissues may be associated with the metabolic interactions between relevant hormones and Se.

We further compared the expression of seventeen insulin signaling related genes and found that dietary Se deficiency down-regulated thirteen signaling related genes in muscle (Fig 5B), six genes in pancreas (Fig 5C) and five genes in liver (Fig 5A), and only three genes (*Foxa2*, *Gcg* and *Irs1*) were up-regulated in liver (Fig 5A). Dietary Se deficiency affected the most of insulin signaling related genes in muscle and the least of genes in pancreas **(**Fig 5**)**, while pancreas had the most affected selenoprotein encoding genes by Se deficiency **(**Fig 4**)**. These divergent patterns of responses may reflect crucial roles of each gene in different tissues. Selenium affected mRNA profiles of key regulators, such as Foxa2, Pdx1, Hnf1A, and Hnf4A, which exert important roles in insulin synthesis and secretion in β cell. Transcription factor Pdx1 is probably the most important regulator for β cell differentiation and survival, as well as expression of the insulin and glucose metabolism related genes [22, 62]. Inactivation of the mouse *Pdx1* gene results in the loss of the β-cell phenotype and maturity onset diabetes [22]. Loss of Hnf1α function in mice leads to abnormal expression of genes involved in pancreatic islet development and metabolism [24]. Hnf4A, as a transcription factor, is essential for human pancreatic β cell function [63]. After entering the nuclear, Hnf4A interacts with Foxo1 and induces the expression of SelP in mammal [64]. Akt1 and Foxo2 play important roles in the regulation of pancreatic cell differentiation and function [65]. In present study, dietary Se deficiency decreased expression of these genes in pancreas and induced pancreatic atrophy in parallel with the impairment in the synthesis and secretion of pancreatic insulin, contributing to the lower plasma insulin.

Muscle and liver are important target organs that insulin exerts its biological function through Pi3k/Akt signaling pathway. We found that the Se deficient diet globally down-regulated thirteen insulin signaling related genes (*Akt1*, *Braf*, *Foxo1*, *Foxa2*, *Ir*, *Irs1*, *Irs2*, *Ins*, *Neurod1*, *Ptpn1*, *Pi3*k, *Slc2A2*, and *Ucp*) in muscle **(**Fig 5B**)**. These affected genes, including *Ir*, *Irs1*, *Irs2*, *Akt1*, *Pi3k*, *Foxo1* and *Foxa2*, encode the major signal proteins in insulin cascade/pathway [66], and the down-regulation of these genes or proteins may compromise insulin sensitivity [67]. A number of studies have shown the ability of selenate to initiate the translocation of glucose transporters to the membrane [17] and to induce phosphorylation of IR [68]. The latter event activates the insulin signaling cascade [69] and allows the interaction of IRS with the regulatory subunit of Pi3k. Forkhead transcription factors of the Foxo family are important downstream targets of Akt, Foxo1 confers insulin sensitivity on glucose 6-phosphatase expression [70]. In present study, dietary Se deficiency globally suppressed expression of insulin signaling related genes, indicating the compromised insulin sensitivity and the potential decreased glucose uptake in skeletal muscle [17], which may contribute to the elevated fasting plasma glucose levels **(**Table 2**)**. In liver, Se deficient diet down-regulated *Irs2*, *Ins*, *Pdx1*, *Ptpn1 and Slc2A2* and up-regulated *Foxa2*, *Gcg* and *Irs1*. *Slc2A2* is an important glucose transporter that responsible for encoding Glut2 [71]. The down-regulation of *Slc2A2* indicated Se deficiency might prevent the uptake of glucose in liver and muscle. *Gcg* encodes glucagon, which modulates blood glucose by enhancing liver gluconeogenesis and glycogen catabolism [72]. The up-regulation of *Gcg* and down-regulation of *Slc2A2* in liver might be partly responsible for the hyperglycemia induced by dietary Se deficiency. Protein-tyrosine phosphatase 1B (PTP-1B) encoded by *Ptpn1* is a major protein-tyrosine phosphatase that regulate of insulin action and other signaling pathways [73]. PTP-1B stabilizes tyrosine phosphorylation of insulin receptor substrate Irs1 and affects the downstream of insulin signaling cascade [74]. In present study, the down-regulation of *Ptpn1* in liver and muscle might imply the suppression of the insulin signal transduction in those tissues by Se deficiency.

## Conclusions

This study indicates that dietary Se deficiency suppresses growth performance of broilers, induces classical Se deficiency disease, nutritional pancreatic atrophy, and decreases antioxidant activities. Dietary Se deficiency globally down-regulate expression of selenoprotein encoding- and insulin signaling related- genes in pancreas, liver and muscle, resulting in dyslipidemia, hypoinsulinemia and hyperglycemia in chicken. The global changes are likely to be associated with the pancreatic atrophy caused by Se deficiency as well as due to Se deficiency *per se*. Our results indicate that dietary Se plays an important role in maintaining insulin function through the regulation of selenoprotein encoding genes in corresponding tissues.

## Supporting information

S1 Table Composition of basal diet (as fed basis). (DOCX)

S2 Table Primers used for the Q-PCR of the target and reference genes. (DOCX)

S1 Fig Photographs of the broilers with Se deficiency symptoms. (A), (B) and (C) represent three different birds, all sub-panels bearing same letter (A1/A2, B1/B2 and C1/C2) are the pictures of the same bird but in different view.
(TIF)

S2 Fig Autopsy of the broiler fed on Se deficient and control diet at fifth week. Control birds displaying normal healthy tissue (A) as against exudative diathesis (greenish, gelatinous edema, with subcutaneous hemorrhage) of severe (B), and less severe grade (C, D) in Se-deficient birds. Arrow indicates the appearance of typical greenish, gelatinous edema, or hemorrhage.
(TIF)
