# Supplementary material for: Pancreatic atrophy caused by dietary selenium deficiency induces hypoinsulinemic hyperglycemia via global down-regulation of selenoprotein encoding genes in broilers
Source: PLoS One. 2017 Aug 1;12(8):e0182079. doi: 10.1371/journal.pone.0182079 (PMC5538751; doi:10.1371/journal.pone.0182079)
Supplement: S1 Table — (DOCX) [file pone.0182079.s001.docx]

**S1 Table.** Composition of basal diet (as fed basis)^1^

| Ingredient | Content, *g/kg* |
| --- | --- |
| Corn | 789.10 |
| Roasted soybean | 170.00 |
| CaCO_3_ | 12.05 |
| CaHPO_4_ | 20.00 |
| L-lys·HCl | 3.50 |
| DL-Methionine | 1.80 |
| Threonine | 2.60 |
| Tryptophan | 0.58 |
| Salt | 3.00 |
| Choline chloride | 2.00 |
| premix^2^ | 5.00 |

^1^The Selenium content of basal diet is less than 0.02 ppm.

^2^The premix consist of mineral and vitamin. Provided (per kg of diet): retinyl acetate, 8000IU; cholecalciferol, 1000IU; tocopheryl acetate, 10 mg; menadione, 0.5 mg; thiamine, 2 mg; riboflavin, 8 mg; D-pantothenic acid, 10 mg; niacin, 35 mg; folic acid, 0.55 mg; cyanocobal-amine, 0.01 mg, manganese 80 mg; iodine, 0.35 mg; iron ,80 mg; Copper, 8 mg; zine, 80 mg; colistin sulfate, 5 mg; flavomycin, 12 mg.
