# Supplementary material for: Pancreatic atrophy caused by dietary selenium deficiency induces hypoinsulinemic hyperglycemia via global down-regulation of selenoprotein encoding genes in broilers
Source: PLoS One. 2017 Aug 1;12(8):e0182079. doi: 10.1371/journal.pone.0182079 (PMC5538751; doi:10.1371/journal.pone.0182079)
Supplement: S2 Table — (DOCX) [file pone.0182079.s002.docx]

**S2 Table.** Primers used for the Q-PCR of the target and reference genes

| **Gene** | **Accession number** | **Primer pairs (5' to 3' direction)** |
| --- | --- | --- |
| **Housekeeping control genes** | | |
| *ACTB* | NM_205518.1 | F：ACCTGAGCGCAAGTACTCTGTCT  R：CATCGTACTCCTGCTTGCTGAT |
| *GAPDH* | NM_204305.1 | F：TTGGCATTGTGGAGGGTCTT  R：GGGCCATCCACCGTCTTC |
| **Selenoprotein genes** | | |
| *Gpx1* | NM_001277853.1 | F：ACGGCGCATCTTCCAAAG  R：TGTTCCCCCAACCATTTCTC |
| *Gpx2* | NM_001277854.1 | F：CCCATCGCCAAGTCCTTCTA  R：CACCTTCTCCCCCTGCAA |
| *Gpx3* | NM_001163232.2 | F：GGCTTCCCCTCCAACCAA  R：GCAGGGAGGATCTCGGAGTT |
| *Gpx4* | NM_204220.1 | F：CTTCGTCTGCATCATCACCAA  R：TCGACGAGCTGAGTGTAATTCAC |
| *Txnrd1* | NM_001030762.2 | F：TACGCCTCTGGGAAATTCGT  R：CTTGCAAGGCTTGTCCCAGTA |
| *Txnrd2* | NM_001122691.1 | F：GCTCTTAAAGATGCCCAGCACTAC  R：GAACAGCTTGAGCCATCACAGA |
| *Txnrd3* | NM_001122777.1 | F：CCTGGCAAAACGCTAGTTGTG  R：CGCACCATTACTGTGACATCTAGAC |
| *Dio1* | NM_001097614.1 | F：GGGCGAAAAGAGCAGAATGA  R：GTGGGACCCCAGTTTTCGT |
| *Dio2* | NM_204114.3 | F：GGCTGACTGCATGGACAACA  R：TGCACACTCGCTCAAATGAAAC |
| *Dio3* | NM_001122648.1 | F：GACCGGAGGGCTACAAGATCT  R：TCTGGAGCCGGGTTTTGTAC |
| *Seli* | NM_001031528.2 | F：TGCCAGCCTCTGAACTGGAT  R：TGCAAACCCAGACATCACCAT |
| *Selk* | NM_001025441.2 | F：GAAGAGGGCCTCCAGGAAAT  R：CAGCCATTGGTGGTGGACTAG |
| *Selm* | NM_001277859.1 | F：ACATCCCGCTGTACCATAACCT  R：TCTCCTCCCGGGTCATGTC |
| *Sepn1* | NM_001114972.1 | F：CAGGATCCATGCTGAGTTCCA  R：GAGAGGACGATGTAACCCGTAAAC |
| *Selo* | NM_001115017.1 | F：CCAGCGTTAACCGGAATGAT  R：ATGCGCCTCCTGGATTTCT |
| *Sepp1* | NM_001031609.2 | F：CCAAGTGGTCAGCATTCACATC  R：ATGACGACCACCCTCACGAT |
| *Sels* | NM_001024734.2 | F：CCGACATGGTGGTAAGAAGACA  R：GCTTGTGCATTCAACTCCTCTTG |
| *Selt* | NM_001006557.3 | F：AGGAGTACATGCGGGTCATCA  R：GACAGACAGGAAGGATGCTATGTG |
| *Selu* | NM_001193518.1 | F：TTGGAGCATCGTGAGAAAGAATT  R：CGGCAGCTTCAAGGACAGA |
| *Sepw1* | NM_001166327.1 | F：TGGTGTGGGTCTGCTTTACG  R：CCAAAGCTGGAAGGTGCAA |
| *Selx* | NM_001135558.1 | F：TGGCAAGTGTGGCAATGG  R：GAATTTGAGCGAGCTGCTGAAT |
| *Sep15* | NM_001012926.2 | F：ACTTGGCTTCTCCAGTAACTTGCT  R：GCCTACAGAATGGATCCAACTGA |
| *Sephs2* | BG711010.1 | F：CCGAGTGCGACAACATGCT  R：GCTCCTCGTCCGTCATCTTCT |
| **Insulin signaling related genes** | | |
| *Akt1* | NM_205055.1 | F：CACGCACTTTGTCACCTGAAG  R：CCGCCTCCTAACCTTTGCTT |
| *Braf* | NM_205302.1 | F：CACAGATTCTTGCCTCCATTGA  R：TGCACTGCGGTGAATTTTTG |
| *Foxo1* | NM_204328.1 | F：TCTGGTCAGGAGGGAAATGG  R：GCTTGCAGGCCACTTTGAG |
| *Foxa2* | NM_204770.1 | F：GGGCCCGTAACGAACAAAA  R：AAGAAGTCTCTCCGGCCAAAG |
| *Gcg* | NM_205260.3 | F：CAGACGAGCTCAGGATTTTGTG  R：CCTGTCCTTGTTGGCCATTT |
| *Hnf1A* | NM_001030668.2 | F：CACAGGCACCAGCGATACAT  R：CGAGGGCCAGACTGAAGGT |
| *Hnf4A* | NM_001030855.1 | F：CGACCCAGATGCCAAAGG  R：GGTACCGCATCCGCTTGAT |
| *Ir* | XM_001233398.3 | F：TGAGACCCGACGCTGAGAATA  R：GCCATCTGGATCATTTCTCTCAGT |
| *Ins* | NM_205222.3 | F：CCAGCAGGAGGAATACGAGAA  R：ACGTGTTATGGCAGCATTGC |
| *Irs1* | NM_001031570.1 | F：CGGTCAGTCTGTCGTCCAGTAG  R：GGAACCAGACACCGAAGCA |
| *Irs2* | XM_425588.4 | F：GCGACCAGTACGTGTTCATGA  R：AGAACCGCTCCGCACATC |
| *Neurod1* | NM_204920.1 | F：TCAGCATCTACGGCAACTTCTC  R：TGGTGAAGGCGTAGCTATTGTC |
| *Pdx1* | XM_001234635.2 | F：GAGCTGGCCGTCATGTTAAAC  R：CCATTTCATCCGTCGGTTCT |
| *Ptpn1* | NM_204875.1 | F：CCAGAGTCTCCTGCTTCATTCC  R：GGGTTAAGCGAGCCAGATTCT |
| *Pi3k* | NM_204658.1 | F：CCTCGGAATTGGAGACAGACA  R：ATGCCACCCGTCTCCTTGT |
| *Slc2A2* | XM_004939879.1 | F：CACACTATGGGCGCATGCT  R：ATTGTCCCTGGAGGTGTTGGT |
| *Ucp* | NM_204107.1 | F：CCTACGACCTCATCAAGGACACA  R：GAAGGCAGCCACGAAGTGA |
