# Supplementary figures and images for: Pancreatic atrophy caused by dietary selenium deficiency induces hypoinsulinemic hyperglycemia via global down-regulation of selenoprotein encoding genes in broilers
Source: PLoS One. 2017 Aug 1;12(8):e0182079. doi: 10.1371/journal.pone.0182079 (PMC5538751; doi:10.1371/journal.pone.0182079)

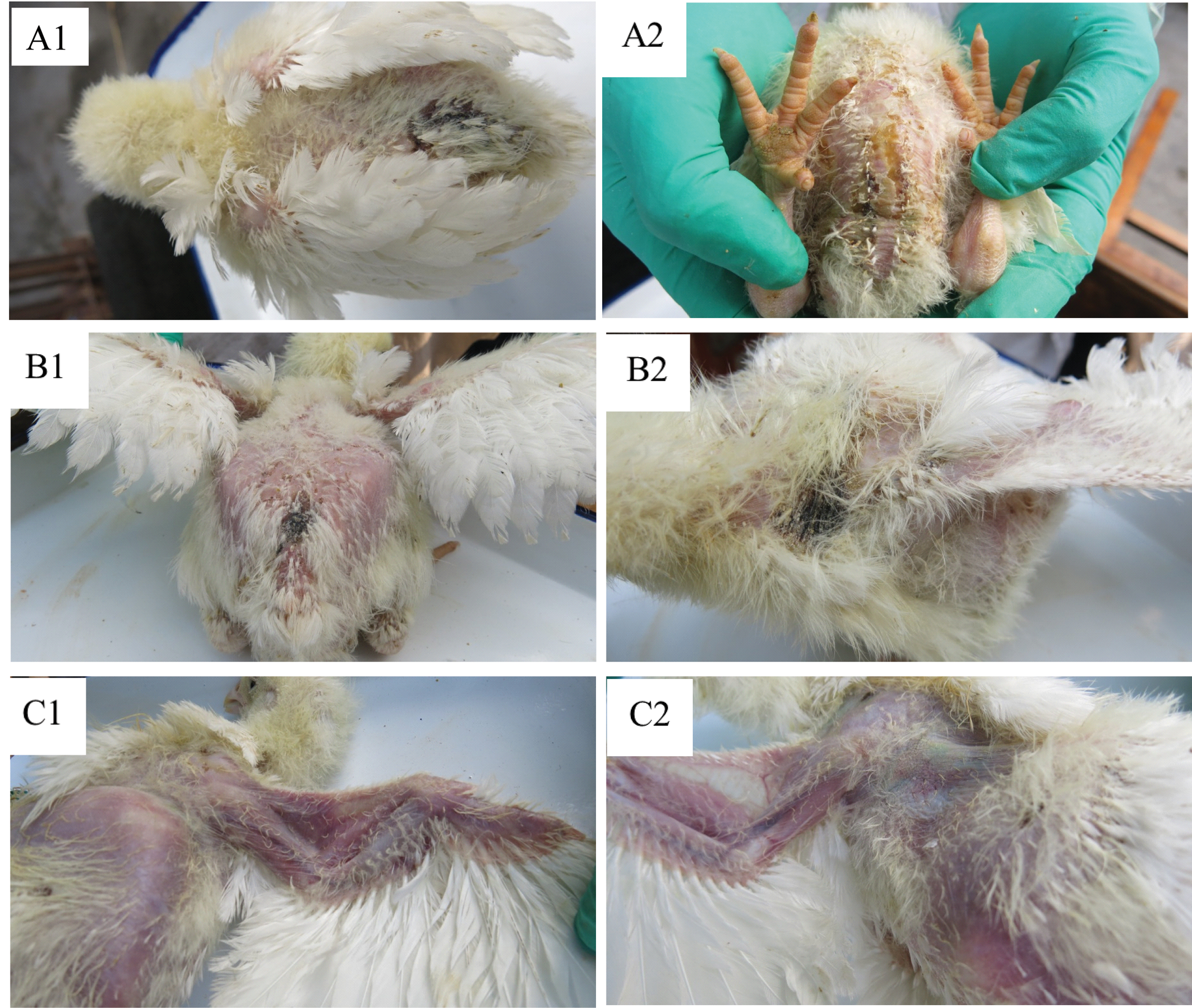

Supplement: S1 Fig — (A), (B) and (C) represent three different birds, all sub-panels bearing same letter (A1/A2, B1/B2 and C1/C2) are the pictures of the same bird but in different view. (TIF) [file pone.0182079.s003.tif]

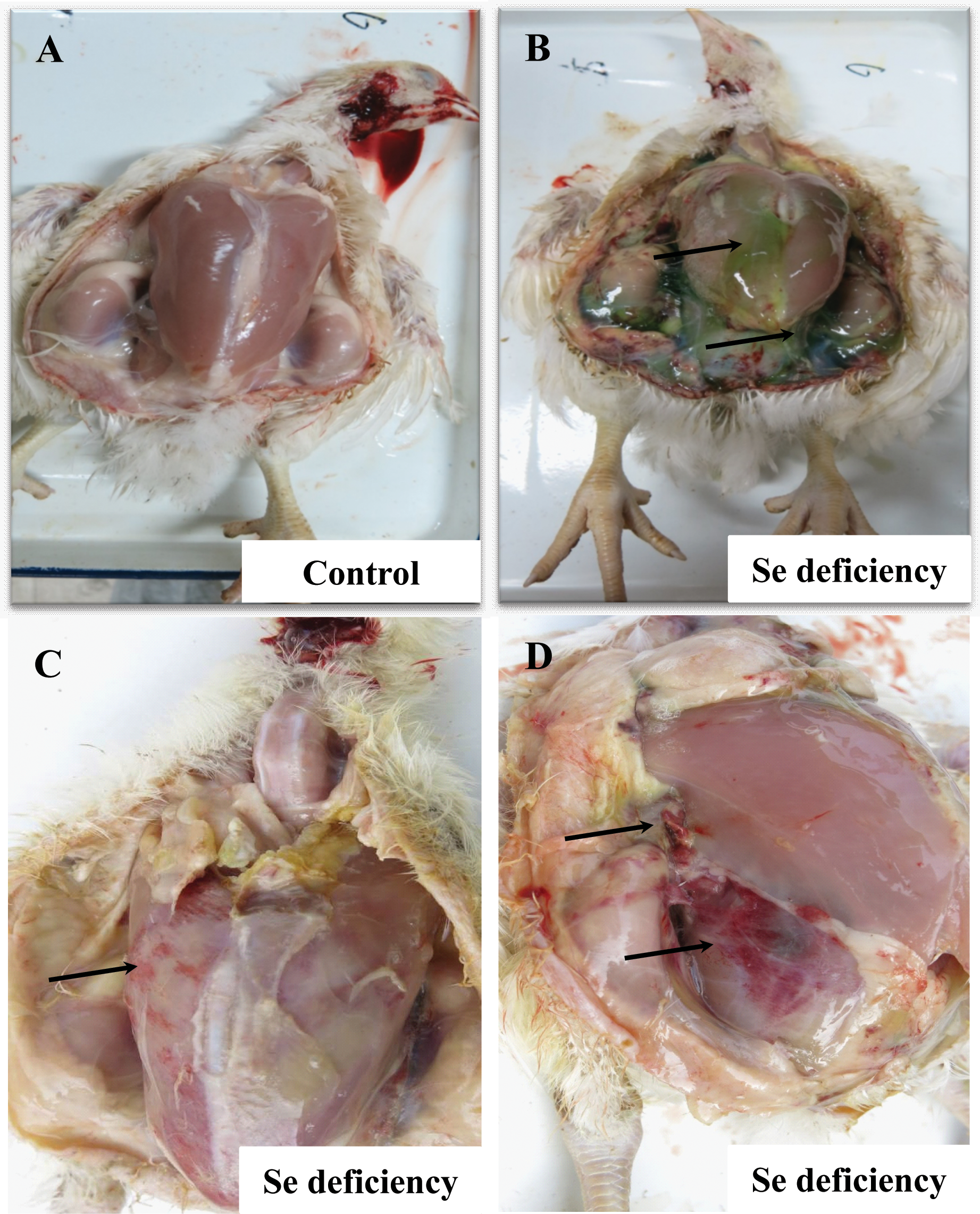

Supplement: S2 Fig — Control birds displaying normal healthy tissue (A) as against exudative diathesis (greenish, gelatinous edema, with subcutaneous hemorrhage) of severe (B), and less severe grade (C, D) in Se-deficient birds. Arrow indicates the appearance of typical greenish, gelatinous edema, or hemorrhage. (TIF) [file pone.0182079.s004.tif]
